# Modified Lipids and Lipoproteins in Chronic Kidney Disease: A New Class of Uremic Toxins

**DOI:** 10.3390/toxins8120376

**Published:** 2016-12-16

**Authors:** Nans Florens, Catherine Calzada, Egor Lyasko, Laurent Juillard, Christophe O. Soulage

**Affiliations:** 1 CarMeN, INSERM U1060, INRA U1397, INSA de Lyon, Université Claude Bernard Lyon 1, University of Lyon, F-69621 Villeurbanne, France; catherine.calzada@insa-lyon.fr (C.C.); egor.lyasko@new.ox.ac.uk (E.L.); laurent.juillard@univ-lyon1.fr (L.J.); christophe.soulage@insa-lyon.fr (C.O.S.); 2 Hospices Civils de Lyon, Department of Nephrology, Hôpital E. Herriot, F-69003 Lyon, France

**Keywords:** uremic toxin, oxidative stress, lipid, lipoprotein

## Abstract

Chronic kidney disease (CKD) is associated with an enhanced oxidative stress and deep modifications in lipid and lipoprotein metabolism. First, many oxidized lipids accumulate in CKD and were shown to exert toxic effects on cells and tissues. These lipids are known to interfere with many cell functions and to be pro-apoptotic and pro-inflammatory, especially in the cardiovascular system. Some, like F2-isoprostanes, are directly correlated with CKD progression. Their accumulation, added to their noxious effects, rendered their nomination as uremic toxins credible. Similarly, lipoproteins are deeply altered by CKD modifications, either in their metabolism or composition. These impairments lead to impaired effects of HDL on their normal effectors and may strongly participate in accelerated atherosclerosis and failure of statins in end-stage renal disease patients. This review describes the impact of oxidized lipids and other modifications in the natural history of CKD and its complications. Moreover, this review focuses on the modifications of lipoproteins and their impact on the emergence of cardiovascular diseases in CKD as well as the appropriateness of considering them as actual mediators of uremic toxicity.

## 1. Introduction

Chronic kidney disease (CKD) is associated with an increased risk of cardiovascular disease as these patients develop accelerated atherosclerosis [1,2,3,4,5,6,7]. The main mechanisms underlying this increased CV risk in this population are oxidative stress [8], accumulation of uremic toxins [9], dyslipidemia and phosphocalcic metabolism disorders. Enhanced oxidative stress and uremic environment can strongly modify circulating lipids and lipoproteins leading to profound alterations of their biological properties. Indeed, lipid peroxidation by-products such as malondialdehyde (MDA) are increased and are negatively correlated with the glomerular filtration rate in CKD. Large amounts of oxidized lipids, such as F2α-isoprostanes are associated with CKD progression [10].

Renal dysfunction is also associated with many perturbations in lipoprotein metabolism leading to dyslipidemia and accumulation of atherogenic particles [11]. Lipoprotein metabolism is complex and is associated with multisite regulations (involving liver, colon, plasma, macrophages and endothelial cells) that can be individually affected by CKD. Post-translational modifications such as carbamylation, glycation or oxidation particularly affect circulating lipoproteins (both on their protidic or lipidic fraction) leading to altered behaviors in the cardiovascular system. These particular modifications of lipid metabolism in CKD are a novel way of explaining the failure of statins in the prevention of cardiovascular diseases in hemodialysis patients [12,13].

According to the European Uremic Toxin Work Group (EuTox, http://www.uremic-toxins.org/), uremic toxins are defined as accumulated solutes, normally excreted by the kidneys, that interact negatively with biological functions [9]. Even if some lipids and lipoproteins are neither excreted by the kidneys in normal conditions nor accumulated in CKD, their modifications and altered metabolism unambiguously change their interactions with biological functions and especially cardiovascular physiology. This review will explain why in CKD, some lipids and lipoproteins can be considered as uremic toxins.

## 2. Uremic Lipoproteins, Evidences of Toxicity

### 2.1. Dyslipidemia in CKD, A Unique Phenotype

CKD is associated with dyslipidemia associating hypertriglyceridemia, elevated LDL cholesterol, an accumulation of ApoB containing lipoproteins, increased concentrations of lipoprotein(a) particles and low HDL levels [14,15]. Many recent reviews analyzed this dyslipidemia in detail [11,14,16,17,18]. Dyslipidemia in CKD is unique for many reasons. First, cardiovascular (CV) diseases are the leading cause of mortality in CKD patients. Number of cardiovascular events has been strongly correlated with GFR decline [1] and despite constant improvement of renal suppletion therapies, such as hemodialysis, this cardiovascular mortality remains at the forefront [19]. Traditional strategies for cardiovascular prevention, including the prescription of statins, failed in some CKD populations. Even if post hoc analysis of large prospective studies sketched a potential benefit in early stages of CKD [20,21,22], this positive effect is diminished in advanced stages (4 and 5), either on intima/media thickness [23] or cardiovascular mortality and related events, as shown by 4D [12] and AURORA [13] studies. Recent meta-analysis from the Cochrane Collaboration confirmed this observation in dialysis patients [24] but also suggested its interest for CKD patients who did not require hemodialysis [25] or transplant recipients [26]. However, beyond its effects on CV mortality, statins exhibited beneficial effects for impeding renal failure progression [27,28]. Indeed, statins can modulate intracellular pathways of inflammatory and fibrogenic responses and inhibit the proliferation of mesangial and renal tubular epithelial cells [27,29]. Moreover, recent data corroborate their importance in lipid control to prevent the progression of CKD. The increase of one standard deviation of TG level and TG/HDL-cholesterol ratio was correlated with an increased risk of developing CKD. Additionally, increases of HDL-cholesterol level, LDL-cholesterol/ApoB and HDL-cholesterol/ApoAI ratios seemed to be protective [30].

### 2.2. Very Low Density, Intermediate Density Lipoproteins (VLDL, IDL) and Chylomicrons

An earlier report suggested that triglyceride-rich lipoproteins (TGRL), including chylomicrons, VLDL and their remnants, accumulate in CKD [31]. Okubo et al. found that ApoB48 levels, composed of chylomicrons and their remnants, are inversely correlated with GFR levels and increased proteinuria [32]. ApoB48 levels were also found elevated in ESRD diabetic patients [33]. In transplantation, ApoB gene polymorphism was associated with poor cardiovascular outcomes in patients presenting deletion of a part of ApoB signal peptide [34].

TGRL, chylomicrons and VLDL, deliver lipids to peripheral cells. After delivering their triglycerides cargos, these lipoproteins are converted into IDL and LDL or are removed by the liver. Chylomicrons and nascent VLDL need apolipoprotein C and E (ApoC, E) for their maturation. These proteins are delivered by HDL-2. In CKD, HDL metabolism is impaired and HDL-3 are not maturated into HDL-2 due to a lecithin-cholesterol acyl-transferase (LCAT) deficiency [35,36,37]. ApoE and ApoC are necessary for binding and activation of lipoprotein lipase (LPL) respectively and such defect leads to a reduced release of triglycerides in peripheral tissues and leads to an accumulation of TGRL. Moreover, significant evidence showed that peripheral LPL is lacking in CKD [38,39,40].

**Figure 1:**
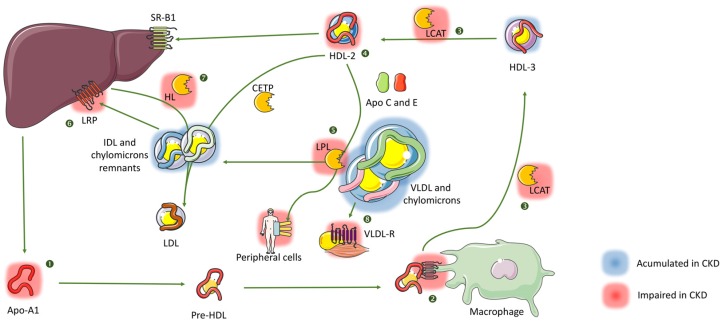
Main modifications of lipoprotein metabolism induced by chronic kidney disease (CKD). CKD induces a deep modification in lipoprotein metabolism resulting in the accumulation of pro-atherogenic particles such as intermediary density lipoprotein (IDL) and triglyceride-rich lipoproteins (TGRL). Main modifications are listed below: In CKD, ApoA1 and A2 levels are decreased resulting in low level of circulating high density lipoprotein (HDL) (❶). In CKD, modifications of ApoA1 decrease HDL binding to macrophages and participate in the observed impaired cholesterol efflux (❷). Nascent HDL are transformed into discoid HDL-3 and then spherical HDL-2 enriched in cholesterol by the action of lecithin-cholesterol acyltransferase (LCAT). In CKD, LCAT level and activity are impaired (❸), leading to the accumulation of HDL-3 and reduced level of HDL-2 (❹). Thus, low HDL-2 concentration result in less transfer of triglycerides from TGRL to HDL-2 by cholesterol-ester transfer protein (CETP). Moreover, HDL-2 fail to enrich very-low density lipoprotein (VLDL) and chylomicrons with ApoC and E, essential for the binding and activation of lipoprotein lipase (LPL) respectively and such defect, associated with evidence of peripheral LPL lacking in CKD, leads to a reduced release of triglycerides into peripheral tissues and leads to an accumulation of TGRL (❺). IDL and remnants accumulate in CKD because of a down-regulation of LDL receptor protein (LRP) (❻), the lower level of CETP (❹) and the down-regulation of hepatic lipase (HL) expression (❼). A part of VLDL accumulates because of the down-regulation of the VLDL-receptor (VLDL-R) in myocytes and adipocytes (❽). Abbreviations: refer to abbreviation section.

In normal conditions, VLDL and chylomicrons are transformed into IDL and chylomicrons remnants after lipolysis in peripheral tissue. Then, part of IDL and remnants are removed by the liver via LDL receptor protein (LRP) that has been found to be downregulated in CKD [41]. The other part of IDL is transformed into LDL by the removal of their triglycerides by the hepatic lipase and enrichment in cholesteryl esters from HDL-2 by cholesteryl-ester transfer protein (CETP). As previously described, lack of HDL-2 impedes this phenomenon and leads to the accumulation of highly pro-atherogenic IDLs [42]. Moreover, there is a downregulation of hepatic lipase expression in CKD [43,44,45]. A part of VLDL is removed by their binding on VLDL-receptor in myocytes and adipocytes. The expression of this receptor is also down-regulated in CKD [46,47] (Figure 1).

### 2.3. Low Density Lipoproteins (LDL)

As LDL remains the main target of cardiovascular prevention strategies, their metabolism in CKD was the focus of numerous works. Patients in early stages of CKD commonly exhibit elevated LDL-cholesterol level [48]. Uncommonly, ESRD patients and particularly HD patients have normal or reduced LDL cholesterol and total cholesterol levels and interestingly, an inversed association has been found between cholesterol level and mortality in those patients [49,50,51]. Beyond LDL-cholesterol level itself, CKD leads to various structural modifications of lipids and proteins that make up LDL particles.

Oxidized-LDLs (oxLDL) result from the action of enzymatic and non-enzymatic pathways of oxidative stress. Several levels of oxidation from electronegative (minimally modified) to extensively oxidized LDL can coexist in the bloodstream and lead to the activation of several pathways involved in atherosclerosis through their binding to scavenger receptors [52]. In CKD, there is substantial evidence that those oxLDL accumulate, especially in HD patients [53,54,55,56]. oxLDL concentration was shown to increase after an HD session [56]. Moreover, oxLDL are correlated with left ventricular hypertrophy in pediatric HD patients [57] and with the intensity of peripheral arterial disease [58]. Oxidized epitopes of LDL can activate immunity and then lead to the formation of antibodies directed against oxLDL. OxLDL/antibodies against oxLDL ratio was also correlated with carotid atherosclerosis and cardiovascular events in HD patients [59].

As stated above, CKD is associated with an enhanced MPO activity that plays a substantial role in the generation of post translational modification derived products (PTMDPs). Indeed, serum MPO levels have been correlated with mortality in an HD cohort [60] and with oxLDL levels [61]. MPO can promote LDL modifications through several mechanisms. First, reaction between HOCl generated by MPO and tyrosine residues of ApoB100 creates 3-chlorotyrosine, found in atherosclerotic lesions [62] and well-known for their pro-atherogenic properties through their binding with lectin-like oxidized LDL receptor 1 (LOX-1) [52,63]. Interestingly, 3-chlorotyrosine levels were found to be higher in HD patients compared to healthy volunteers [64]. Secondly, MPO can also catalyze the generation of reactive nitrogen species (RNS) and create pro-atherogenic nitrosilated-LDL [65]. Thirdly, MPO catalyzed the addition of thiocyanate to the LDL (derived from the decomposition of urea) and leads to the formation of carbamylated-LDL (cLDL) [66,67]. These cLDL have potent pro-atherogenic effects such as the transformation of macrophages into foam cells [68] through their binding to the pro-atherogenic CD36 receptor [69] (upregulated in CKD [70]) and associated with endothelial toxicity [71,72] and platelet aggregation [73] through LOX-1 [74]. cLDL levels are raised by chronic uremia and were linked with atherosclerosis in CKD [75,76].

### 2.4. High Density Lipoproteins (HDL)

Accelerated atherosclerosis in CKD patients and relative failure of statins in advanced stages of CKD demonstrate that traditional cardiovascular risk factors are not at the forefront. A recent focus on HDL quality rather than quantity revealed a potential role of HDL dysfunction in the set-up of cardiovascular disease in CKD.

HDL metabolism is impaired in CKD. In normal physiological conditions, ApoA1 and A2 are released into the circulation by the liver. These proteins are loaded with cholesterol and phospholipids to form nascent HDL. In CKD, ApoA1 and A2 levels are decreased [77,78]. Then, nascent HDL binds to the ABCA-1 receptor on circulating macrophages and activates cholesterol ester hydrolase allowing their loading with cholesterol. AcetylCoA acyl transferase (ACAT) limits this reverse efflux of cholesterol from macrophages by catalyzing the esterification of intracellular cholesterol. In CKD, modifications of ApoA1 limit HDL binding on macrophages [79] and upregulation of hepatic ACAT-2 in CKD [37,80] participates in the observed impaired cholesterol efflux. After its uptake, free cholesterol is esterified into cholesteryl esters by lecithin cholesterol acyl transferase (LCAT), which is transferred to the core of the lipoprotein. Nascent HDL are then transformed into discoid HDL-3 and then into spherical HDL-2 enriched in cholesterol. In CKD, LCAT level and activity are impaired [35,36,37], leading to the accumulation of HDL-3 and reduced level of HDL-2, especially in HD patients [81]. The latter, in normal conditions, enrich VLDL and chylomicrons with ApoC and E, essential for their normal metabolism (see above). Moreover, cholesterol ester transfer protein (CETP) transfers triglycerides in exchange for cholesterol from TGRL to HDL and LDL resulting in TG-enriched HDL-2 and LDL species. There is no evidence of deficit of CETP in CKD but as HDL-2 level is lowered, its activity may suffer. Then, HDL is cleared from their cholesterol content by their binding with hepatic SR-B1 receptor and the cycle starts again (Figure 1).

Beyond a dysfunctional metabolism, HDL is also a major target for oxidative stress and post-translational modifications. As stated above, CKD leads to a modification of ApoA1, impairing its binding on ABCA-1 [79]. As MPO activity is enhanced in CKD, MPO-modified ApoA1 results in decreased reverse cholesterol efflux and a reduced binding with ABCA-1 receptor [82,83]. Moreover, MPO leads to the increased formation of 3-chlorotyrosine, an oxidation product of MPO, in HDL and impairs LCAT and paraoxonase activities and then anti-inflammatory proprieties of HDL [84]. MPO-modified HDL are also potentially involved in the generation of foam cells in atherosclerotic lesions through the activation of SR-B1 in macrophages [85] and increase of pro-inflammatory secretion activity and adhesion molecules expression in endothelial cells [86]. These MPO-modified HDL exhibit impaired anti-apoptotic properties in endothelial cells [86]. Interestingly, high levels of oxidized HDL are correlated to increased cardiovascular mortality in HD patients [87] as well as to HDL anti-inflammatory index elaborated by Kalantar-Zadeh et al. [88]. Recent analysis of HDL proteome showed an association of acute phase protein serum amyloid A with CKD-HDL that may participate in impaired biological functions [89,90].

Normal HDL are known to be anti-atherogenic thanks to several properties [17]. First, HDL induces a reverse cholesterol transport from circulating macrophages. This property is well known to be atheroprotective [91]. In CKD, this function is dramatically impaired as CKD-HDL have reduced capacities of inducing this efflux [81,89,92,93,94]. Even in a pediatric CKD cohort, the deeper was CKD, the lower was the cholesterol efflux [95]. Moreover, restoration of renal function by transplantation is associated with an enhancement of these capacities without retrieving normal-HDL levels [92]. Nevertheless, Kopecky et al. sowed the seeds of doubt by showing that cholesterol efflux levels capacity in diabetic HD patients is not a prognostic marker of cardiovascular events [96].

Secondly, normal HDL increases the production of nitric oxide through the activation of eNOS in endothelial cells resulting in a vasorelaxant phenotype. In CKD, evidences show that HDL from CKD children lose their protective effect as the production of NO by endothelial cells is significantly reduced with HDL [95]. CKD-HDL can probably induce the uncoupling of eNOS from endothelial cells as the superoxide production in endothelial cells is significantly enhanced with CKD-HDL [95].

Thirdly, normal HDL inhibits the expression of adhesion molecules such as intercellular adhesion molecule-1 (ICAM-1) and vascular adhesion molecule-1 (VCAM-1), which prevent the attachment of circulating monocytes to endothelial cells. In CKD, HDL promotes an enhanced expression of VCAM-1 and ICAM-1 on endothelial cells [93,95]. Moreover, CKD-HDL upregulates the expression of pro-inflammatory mediators such as monocyte chemoattractant protein-1 (MCP-1), interleukin-1ß (IL-1ß) and tumor necrosis factor α (TNF-α) [93,94].

Normal HDL exhibit anti-apoptotic effects on endothelial cells through the downregulation of caspase-3 activity [97]. CKD-HDL inhibits endothelial cell proliferation [93]. Finally, normal HDL has anti-oxidative properties thanks to PON1 and GPX enzymes on its surface. PON1 was firstly recognized as a hydrolytic enzyme for various toxic organophosphates. Mainly expressed in the liver and the kidney, this enzyme exhibited anti-oxidant properties against lipid peroxidation as it binds to HDL and in a minor part to VLDL [98]. Thus, this enzyme is considered as the main anti-oxidant enzyme bound to HDL. PON1 activity is lowered in CKD [99,100,101]. GPX is another important anti-oxidant enzyme at HDL surface. Its activity and expression on HDL are lowered in CKD [35].

### 2.5. Lipoprotein A (Lp(a))

Lp(a) is composed of a LDL-like particle bound with an apolipoprotein(a) (Apo(a)) on lysine residues of ApoB100. Apo(a) is secreted by the liver and contains a repetition of kringle-IV units. Genetic variants of Lp(a) and its concentration have been deeply correlated with coronary heart disease and cardiovascular morbidity [102,103,104,105]. As its concentration is closely associated with OxPL/ApoB, Lp(a) may play a role in OxPL clearance even though its role(s) still remain unclear. Lp(a) levels were found to be risk predictors of all-cause mortality in HD patients [106]. Lp(a) accelerates atherosclerosis in a mouse model of CKD [107]. Moreover, Lp(a) clearance is partly done by the kidney explaining why its clearance is lowered in hemodialysis patients even if its generation does not seem to be higher than in healthy subjects [108]. However, in Tzanatos et al. study, Lp(a) levels seems to be increased after an HD session [109] while no change was found in Bossola et al. study [56]. As HD seems to be ineffective for Lp(a) clearance, it can explain higher levels in ESRD patients as its generation is not increased in these patients. Unlike hemodialysis, nephrotic syndrome exhibits enhanced secretion of Lp(a) by the liver [110]. In a nutshell, CKD exhibits higher levels of Lp(a) than in healthy subjects (Table 1) and it accumulates with CKD [111] severity while it decreases with renal transplantation [112], Lp(a) is a prototype candidate to be classified as a uremic toxin.

## 3. Oxidative Stress/Non-Oxidative Modifications of Lipids and Lipoproteins in CKD

### 3.1. Oxidative Stress, Lipid Peroxidation and Antioxidant Defenses

Oxidative stress is defined as a lopsided balance of the pro/anti-oxidant state in favor of the pro-oxidant [129]. The origin of this stress is the formation of reactive oxygen species (ROS) like superoxide anion O2^•−^, hydroxyl radical ^•^OH or hydrogen peroxide H_2_O_2_. Major part of reactive oxygen species in our organism is produced by the mitochondrial respiratory chain [130], the NADPH oxidase [131,132] and 5-lipooxygenase enzyme [133]. Other enzymes such as xanthine oxidase or NO synthase [134] can also provide ROS in pathological conditions. ROS, by many intertwined reactions, produce free radicals in the presence of transition metal ions (Fe^2+^, Cu^2+^) or carbon-composed molecules like proteins, nucleic acids or lipids. Nitric oxide (NO) can also yield free radicals often referred to as reactive nitrogen species (RNS). NO is generated by NO synthases and plays many roles in the regulation of vascular tone, permeability and platelet adhesion. NO can rapidly react with O2^•−^ to generate a more oxidized form of a nitric product: the peroxinitric ion (ONOO^−^). The latter can easily react with proteins, lipids or nucleic acids, resulting in oxidized or nitrosylated forms.

As previously described, lipids can be affected by oxidative stress. First step of lipid peroxidation is the reaction of a free radical with a poly-unsaturated fatty acid (L). This reaction results in the formation of a lipid radical L^•^. This radical can react with oxygen and create lipid peroxyl radicals (LOO^•^). From this point, LOO^•^ can react with other lipids and create new lipid radicals and lipid hydroperoxide (LOOH). The degradation of lipid hydroperoxide provides new lipid radicals (LO^•^, LOO^•^) and aldehydes as stable end-products of lipid peroxidation process (malondialdehyde or MDA, 4-OH-2,3-alkenals). F2α-isoprostanes are end-products from the oxidation of arachidonic acid (i.e., 20:4 (n-6)). These three end-products are routinely used for in vivo evaluation of lipid peroxidation level [135,136]. However, there are other products of lipid peroxidation such as oxysterols and oxidized phospholipids that play a substantial role in the onset and progression of atherosclerosis and lipid dysmetabolism [137].

Under physiologic conditions, there is a basal rate of production of ROS as well as a limited production of oxidized molecules [138]. Nevertheless, this production of oxidants is balanced by a complex pattern of antioxidant mechanisms that protect the cells and tissues from oxidative damages. The protection from the ROS damage is permitted by antioxidant enzymes such as superoxide dismutase (SOD) which catalyses the dismutation of O2^•−^ into H_2_O_2_, glutathione peroxidase (GPX) or catalase, which detoxifies H_2_O_2_ and other hydroperoxide containing molecules. Non-enzymatic antioxidants include reduced glutathione (GSH), which allows the scavenging of ^•^OH and acts as a substrate for GPX as well as ascorbic and uric acids that are scavengers of ^•^OH, singlet O_2_ and peroxyls radicals. Ferritin, ceruleoplasmin, transferrin, lactoferrin and metallothionein can also be regarded as antioxidant proteins as they trap transition metal ions and prevent ROS formation from Fenton reaction [133]. To prevent lipid peroxidation, aforementioned antioxidant molecules act together with several liposoluble antioxidants such as tocopherols, ubiquinol, flavonoids and carotenoids. Moreover, the protein paraoxonase-1 (PON1) from HDL is a major anti-oxidant preventing lipoproteins from oxidation although its exact mechanism remains unclear [98]. Some molecules such as tocopherols or curcumin, are considered as chain-breaking antioxidants, corresponding with their ability to intercept intermediary radicals during the lipid peroxidation process and then break the oxidative chain. Finally, albumin can be considered as a major antioxidant protein of the plasma. In fact, serum albumin can bind various ligands such as copper, iron, long chains fatty acids (LCFA), poly-unsaturated fatty acids (PUFAs) and cholesterol and prevent them from oxidative modifications [139]. It can also bind bilirubin [140,141] and inhibit lipid peroxidation as well as prevent damages of α-tocopherol [142] and bind homocysteine. Albumin also contains a reduced cysteine residue (Cys34) which can scavenge hydroxyl radicals [143]. Due to the large amount of albumin in the plasma, it represents the largest amount of thiols available in the circulation [144]. Albumin also scavenges hypochlorous acid (HOCl) responsible for chlorination of proteins mediated by myeloperoxidase.

### 3.2. Oxidative Stress in CKD

Substantial literature is available about the enhanced oxidative stress in CKD [145,146,147,148,149,150,151,152,153,154]. The unbalanced pro-oxidative state appears almost at the onset of CKD and increases as the glomerular filtration rate (GFR) declines [8]. There are multiple reasons for this enhanced oxidative stress and they are often intertwined.

As we will discuss later, antioxidant defenses are lowered in CKD leading to a higher sensitivity to oxidative stress induced by classical cardiovascular risk factors (hypertension, advanced age, diabetes and obesity [155]). Indeed, hypertension is well known to be a major state of oxidative stress [156,157]. Upregulation of NADPH oxidase via the activation of renin-angiotensin system (RAS) is well-recognized as a major provider of ROS in hypertension [157,158,159]. In CKD, RAS has been directly linked with enhanced oxidative stress and CKD progression through the up-regulation of pro-oxidative pathways (NF-kB, NADPH oxidase, cyclooxygenase 2, 12-lipooxygenase) by angiotensin II and its binding to angiotensin-1 receptor [159].

Most antioxidant defenses are lowered in CKD. However, some controversies still exist as many studies produced conflicting results. Total antioxidant status is decreased in CKD [160,161]. Nevertheless, the large range of techniques used for this determination and the numerous confounding factors in CKD make it difficult to interpret [8,162]. The determination of Superoxide dismutase (SOD) activity is prototypical. Some studies found a decreased SOD activity in CKD patient [163,164] while others reported a normal level of activity [165]. Surprisingly, SOD activity was even found to have increased in CKD [166]. As there are several isoforms of SOD (cytosolic, mitochondrial and extra-cellular), assays are difficult to extrapolate between all the different studies. As a matter of fact, SOD activity seems to be correlated with CKD stage and its activity is restored by several interventions in CKD patients (erythropoietin, vitamin E supplementation and kidney transplantation) [167,168]. Similar contradictory observations were reported for catalase activity (e.g., either lowered [169], normal [165,170] or increased [160]). Glutathione activity and concentration are lowered in CKD and are correlated with uremic toxins’ concentrations [171,172] and glomerular filtration rate (GFR). PON1 activity is lowered in CKD [98,99,100]; however, further studies are needed to fully understand its implication on lipid dysmetabolism and lipoprotein dysfunction [97,173] associated with CKD.

### 3.3. Post-Translational Modification Derived Products (PTMDPs) of Lipoprotein and Covalent Modifications of Lipids

**Figure 2:**
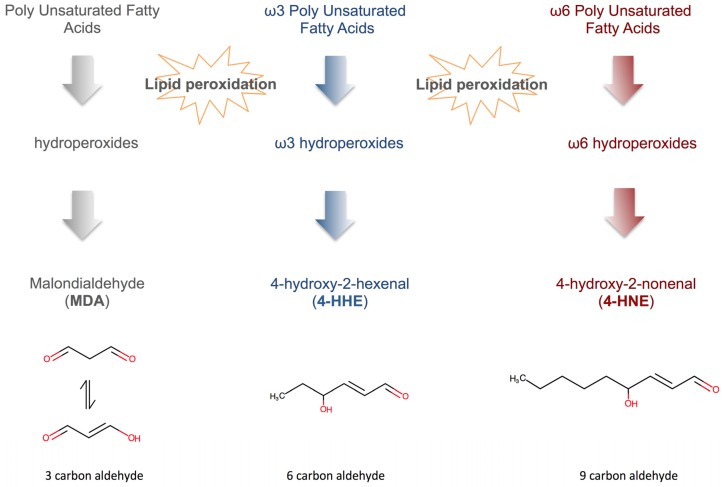
Major reactive lipid aldehydes derived from poly unsaturated fatty acids (PUFAs) oxidation. Malondialdehyde (MDA) results from the oxidation of various polyunsaturated fatty acids containing more than two double bounds. MDA binds with nucleic acids or lysine amino-groups and creates toxic adducts called advanced peroxidation lipid end products (ALEs). On ApoB, these adducts were associated with atherosclerosis. 4-hydroxy-2-nonenal (4-HNE) and 4-hydroxy-2-hexenal (4-HHE) result respectively from the oxidation of ω6 PUFAs and ω3 PUFAs. They can react with proteins by a Michael addition mechanism and create ALEs. These ALEs disrupt several biological functions and lead to the formation of atherosclerosis and foam cells. Abbreviations: refer to abbreviation section.

Proteins and lipids are especially prone to oxidation and their irreversible oxidative modifications lead to a profound alteration of their biological functions. Carbonylation is the addition of compounds made from glycation and lipid peroxidation onto proteins. These residues can react with lysine and arginine residues and then create advanced glycation end products [174] (AGEs) and advanced lipoxidation end products [175] (ALEs). Proteins can also be carbonyled by direct oxidation by ROS [176]. 3-desoxyglucosone, D-arabinose, glyoxal can react with proteins and create AGEs as pentosidine and carboxy-methyl-lysine. Lipid peroxides of polyunsaturated fatty acids (PUFAs) such as 4-hydroxy-2-nonenal (4-HNE) and 4-hydroxy-2-hexenal (4-HHE) can also react with lysine, cysteine and histidine residues of proteins and create ALEs (Figure 2).

AGEs and ALEs have several biological effects involved in atherosclerosis [177]. AGEs have significant effects on lipids as they can make LDL more prone to oxidative modifications [178], increase glycated-LDL uptake from macrophages by scavenger-receptors and accelerate the formation of foam cells [179]. ALEs also exhibit several pro-inflammatory effects and are involved in the progression of atherosclerosis [180]. Both AGEs and ALEs were reported to accumulate in CKD [181,182,183,184,185].

**Figure 3:**
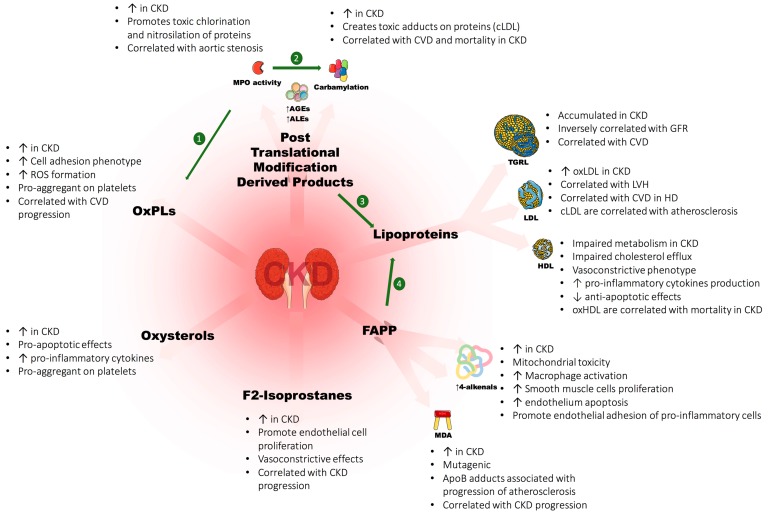
Main effects of oxidized lipids and lipoproteins in chronic kidney disease (CKD). CKD is associated with increased oxidative stress, which promotes covalent modifications of lipids and lipoproteins. Lipid products of this unbalanced metabolism are oxidized phospholipids (oxPLs), fatty acid peroxidation products (FAPPs), oxysterols and F2-isoprostanes. Posttranslational modification derived products (PTMDPs) are the result of an enhanced myeloperoxidase (MPO) activity in CKD, an increased carbamylation and a massive production of advanced glycation end products (AGEs) and advanced lipoxidation end products (ALEs). ALEs are derived from lipid aldehydes issued from peroxidation of fatty acids (FAPPs). MPO catalyzes the nitrosilation on phospholipids to create oxPLs (❶). MPO are also involved in carbamylation process by the addition of thiocyanate on proteic residues (❷). Lipoproteins are also modified in CKD. First, triglyceride-rich lipoproteins (TGRL) have an impaired metabolism leading to their accumulation. Low-density lipoproteins (LDL) exhibit large amount of toxic oxidized (oxLDL) and carbamylated (cLDL) forms in CKD. These modifications lead to impaired functions and promote the progression of cardiovascular disease (CVD) especially in hemodialysis (HD) patients. High density lipoproteins (HDL) are also modified in CKD. Their whole metabolism is impaired and this dysregulation leads to many pro-atherosclerotic effects. MPO and carbamylation are greatly responsible for lipoproteins’ modifications and dysfunctions (❸) so are FAPP products that generate ALEs, especially on apolipoproteins A and B (ApoB) (❹). Abbreviations: refer to abbreviation section.

Carbamylation is the fixation of isocyanic acid, derived from the decomposition of urea on amine groups of proteins. Protein carbamylation is associated with cardiovascular disease [186], mortality in CKD [187,188] and can also affect lipoproteins [75] and promote atherosclerotic complications [68,189] (Figure 3).

Myeloperoxydase (MPO) activity contributes to the formation of chlorinated [64] and nitrosilated [65] proteins or lipids that are correlated with poor cardiovascular outcomes [62,190]. Its activity can also lead to the oxidation and modification of lipoproteins and especially the adduction of thiocyanate produced from the decomposition of urea [67]. In CKD and especially in hemodialysis, this activity is enhanced [60,61,191,192]. Wada et al. showed that MPO expression is associated with aortic stenosis in hemodialysis (HD) patients [193].

## 4. Oxidized Lipids in CKD: Evidences of Toxicity

### 4.1. Cholesterol and Oxysterols

Cholesterol is a major component of cell membranes. Its presence in almost all of the cell membranes makes it a perfect target for ROS. There are four major products of ROS-mediated cholesterol oxidation: 7α,β-OOH-Cholesterol, 7α,β-OH-Cholesterol, 7-oxo-Cholesterol and 5,6-epoxy-Cholesterol. However, there are many other minor forms of oxysterols produced by non-radical pathways (5-α-OOH-Cholesterol, 6α,β-OOH-Cholesterol with singlet oxygen; 5,6β-epoxy-Cholesterol and ozone; 5,6-dichlorocholestane and HOCl from neutrophils) [194].

Oxysterols play a role in the set-up of atherosclerosis as several studies found them in fatty streaks, aortic or atherosclerotic plaques [195,196,197]. Oxysterols exhibited a pro-apoptotic effect on monocytes [198,199], vascular cells (smooth muscle cells [198,200], endothelial cells [198,201]) and hepatocytes [202]. Particularly, 7-oxo-cholesterol and 7α,β-OH-Cholesterol triggered the major toxic effect [198] but other products such as 5,6-ep-oxy-Cholesterol also showed a potential pro-apoptotic effect [201,203]. Other deleterious effects have been shown from various oxysterols including death of macrophages in latter stages of atherosclerosis [204], production of pro-inflammatory cytokines [205,206], LDL oxidation [207] and platelet aggregation [208]. Nevertheless, their role in the modification of cholesterol metabolism still remains unclear [194,209].

Oxysterols are metabolized by the liver and excreted as bile acids. In healthy subjects, oxysterols are found at very low plasma concentrations [210] (Table 1). These concentrations are significantly increased in CKD patients compared to a control population and especially in end-stage renal disease (ESRD) patients undergoing hemodialysis [113,114]. Moreover, Siems et al. reported an increase of their concentration after an HD session [114].

### 4.2. Oxidized Phospholipids

Phospholipids (PLs) are the main components of cell membranes and, as cholesterol, are more exposed to oxidative stress and ROS. They also make up the external layer of lipoproteins and are a preferential site of oxidative and covalent modifications. Oxidized phospholipids can be produced through several pathways. Peroxyl radicals are derived from the free-radical-dependent oxidation of PUFAs esterified into PLs. [211]. Nitration can happen on PLs and generate nitrated-PUFAs and PLs [212]. Halogenation by a direct action of HOCl or HOBr released by neutrophilic and eosinophilic cells can also modify PLs by the addition of halogenide and hydroxyl groups leading to halohydrins (chlorydrins [213] and bromohydrins [214]) residues on PLs. Indeed, MPO and eosinophilic peroxidase activities are increased in CKD [60,61,191,192]. Enzymatic pathways of oxidation are almost always associated with the activity of 12- and 15-lipoxygenases, leading to the formation of hydroperoxides residues on PLs either in cell membranes [215] or lipoproteins [216,217].

These oxidized PLs (Ox-PLs) exhibit several biological effects [218]. First, they play a role in enhanced cell expression of adhesion molecules. Ox-PLs are involved in the activation of β1-integrin/fibronectin [219,220] and P-selectin [221,222] pathways of adhesion. Ox-PLs can also increase the generation of ROS by the elevation of the activity of NADPH oxidase [223]. As PLs are key activators of blood coagulation and platelet activation, Ox-PLs can also modulate this phenomenon. Effects on blood coagulation are mitigated as Ox-PLs can exert opposite effects on several steps of the coagulation cascade even if a pro-coagulation tendency raises from these studies [224,225,226]. Ox-PLs have shown pro-aggregate properties on platelets and vascular cells [227,228,229,230]. Many others effects of Ox-PLs are described in the literature (smooth muscle cells, bone, pro-angiogenic properties…) [211,231].

Part of Ox-PLs biological activity results from their binding to CD-36 receptor (a class B scavenger receptor) [232,233]. This receptor is well-known for its implication in atherosclerosis [69] and its expression is increased in CKD patients [70].

Moreover, high OxPL/ApoB ratio has been related to severe coronary artery disease [102] in patients without CKD (mean level of about 0.20 and 0.15 for respectively <60 years and >60 years population). This ratio has been correlated with the presence and progression of carotid, femoral atherosclerosis and cardiovascular disease for a ratio value higger than 0.088 [103]. Interestingly, in ESRD patients undergoing HD, this mean ratio was found to be at 0.13 [56] (Table 1). Nevertheless, this ratio was not found to be associated with cardiovascular disease in hemodialysis patients [56,115]. Indeed, their concentration decreases after an HD session [56]. This decrease may partially explain the absence of association in HD patients. This ratio has been also associated with renal progression in systemic lupus erythematous patients [234].

### 4.3. Fatty Acid Peroxidation Products (FAPP)

Fatty acids represent an important source of energy in human body physiology. Most of the time, they are derived from triglycerides or phospholipids and can be either saturated or unsaturated. Poly unsaturated fatty acids (PUFAs) are more prone to oxidation, and especially in CKD.

Malondialdehyde (MDA) is the result of polyunsaturated fatty acid oxidation containing more than two double bounds. More than a simple marker of lipid peroxidation, it covalently binds to proteins and nucleic acids, interfering with their normal biological functions. Indeed, MDA binding with nucleic acids can form several toxic adducts [235] and induce frameshift mutations and base-pair substitutions [236] (Figure 2 and Figure 3). Furthermore, MDA can react with lysine amino group and generate lysine-lysine bounds [237]. On ApoB, these bounds were associated with atherosclerosis [238]. MDA can be assayed itself or detected as a derived product of its reaction with thiobarbituric acid (TBA) that produces thiobarbituric acid reactive species (TBARS) [239]. Several studies have shown that MDA is elevated in CKD and represents a good marker of increased oxidative stress [116,165,166,240,241,242,243,244]. Moreover, MDA (or TBARS) levels are correlated with GFR [241], creatinine levels [245] and intensity of renal damages [166]. Surprisingly, data on HD behavior of MDA concentration are controversial. In Kuchta et al. study, it was not affected by HD procedure [162]. In other reports, HD session triggered a significant decrease of the MDA concentration [116,117] but was found to increase in a Nigerian cohort [118]. As an evidence of toxicity and as a significant part of MDA is excreted in the urine [246], EuTox group has already classified MDA as an uremic toxin [9].

Polyunsaturated fatty acids can also generate other reactive lipid aldehydes such as acrolein and 4-hydroxy-2-alkenals [237]. The latter, and particularly, 4-hydroxy-2-nonenal (4-HNE) (a by-product of the peroxidation of n-6 PUFAs) and 4-hydroxy-2-hexenal (4-HHE) (a by-product of the peroxidation of n-3 PUFAs) can react with proteins by a Michael addition mechanism (Figure 2). These adducts can especially bind to histidine, cysteine and lysine residues [247]. These ALEs disrupt several biological functions such as Na+/K+ ATPases [248], mitochondrial functions (potential role in permeability transition [249] and membrane fluidity [250]). These adducts are also found in large amounts in human atherosclerotic lesions [251]. Furthermore, HNE-modified LDL can activate macrophages and increase the up-regulation of class A scavenger receptors involved in the transformation of these cells into foam cells [252]. 4-HNE adducts induce smooth muscle cell proliferation in aortas of rats by the upregulation of ERK1 and ERK2 pathways [253]. 4-HNE can also alter vascular permeability and trigger apoptosis of endothelial cells [254] and promote the adhesion of pro-inflammatory cells to the endothelium [255]. There is some evidence that 4-HNE accumulates in CKD and especially in ESRD patients [256]. In a study by Sommerburg et al., mean level of 4-HNE was 3 fold higher in HD patients compared to healthy controls [257]. Alhamdani et al. showed that alkanals, alkenals and 4-hydroxy-alkenals concentrations are increased in HD patients compared to controls [258] (Table 1). Interestingly, HD procedure tends to reduce the 4-HNE concentration in several reports [116,119,120]. However, as the major part of 4-HNE reacts with proteins and creates stable adducts (ALEs), this observed reduction can over-estimate the decrease of the overall pool of 4-HNE in an HD session. As a major proof of toxicity and as part of alkenals are excreted in the urine [121], EuTox group has already classified 4-hydroxy-2-alkenals as a uremic toxins [9]. To the best of our knowledge, there is only limited data in the literature regarding the levels of 4-HHE in patients although its cytotoxic effect on proximal tubular cells was demonstrated in vitro [259].

### 4.4. F2-Isoprostanes

Arachidonic acid peroxidation generates F2-isoprostanes by a cyclo-oxygenase independent pathway [260,261,262]. Part of these isoprostanes is unesterified but a great majority remains esterified. 8-epi-PGF_2α_ acts as an agonist/antagonist on platelet aggregation via Thromboxane A2 receptor (TxA2-R). Added at high concentrations, it directly induced platelet aggregation via TxA2-R whereas at low concentrations, it inhibited platelet aggregation. Thus, low amount of 8-epi-PGF_2α_ acts as an antagonist of this receptor and inhibits platelet aggregation [263] induced by a TXA2 agonist. F2-isoprostanes promote endothelial cell proliferation and endothelin-1 secretion [264]. Moreover, these compounds have potential vasoconstrictive effects on smooth muscle cells [265]. Intra-arterial infusion of F2-isoprostanes led to a decrease in GFR and these effects were counteracted with TxA2-R antagonists [266]. Additionally, high containing-vitamin E diet in aged rats increased the GFR by 50% and reduced glomerular sclerosis concurrently with a reduction of F2-isoprostanes concentration [267]. Therefore, F2-Isoprostane accumulation may play a role in CKD progression [10], especially since its concentration is closely correlated with GFR [8,268]. In addition, F2-isoprostane concentrations are elevated in HD patients in several studies [119,122,123,124,125] compared with intra-studies control groups and data from the literature [269] (Table 1) making them suitable for classification as uremic toxins and particularly because their concentrations decrease after kidney transplantation [270,271]. Part of these F2-isoprostanes are cleared by the kidney [126,127] but an HD session seems to have no effect on their concentration [119,128].

## 5. Conclusions

CKD is associated with deep modifications in oxidative stress balance, lipid metabolism and turnover, which is responsible for an accumulation of various toxic forms of lipids and lipoproteins. EUtox defined a uremic toxin as an accumulated solute, normally excreted by the kidneys, that interacts negatively with biological functions. As these molecules accumulate in ESRD and exhibit many noxious effects on cell metabolism, CKD progression, cardiovascular system they can be regarded as uremic toxins even if they are not always excreted by the kidneys. Future efforts need to be concentrated on the enhancement of the removal of these lipids and the avoidance of their generation.

## Figures and Tables

**Table 1:** Concentrations of plasma oxidized lipids and lipoproteins in control and CKD patients.

	Normal	CKD	Clearance	HD Behavior	References
**Oxysterols (Total)**			Liver metabolism	Generated during HD session	[113,114]
7-ketocholesterol, nM	32.3 ± 16.7	42.2 ± 30.1 ^δ^			
7β-OH-cholesterol, nM	14.4 ± 7.7	42.6 ± 24.1 ^δ^			
**Oxidized Phospholipids**			Enzymatic detoxification	Reduced after HD session	[56,103,115]
OxPL/ApoB ratio, AU	0.068 ± 0.07	0.138 * ± 0.170 *			
**PUFAs Aldehydes**					[7,9,23,116,117,118] [9,116,119,120,121]
Malondialdehyde (MDA), μg/L	257.7 ± 81.7	388.8 ± 21.6 ^δ^	Enzymatic detoxification Renal excretion	Controversial (decrease, no change and increase)	
4-hydroxy-decenal, μg/L	10.3 ± 7.1	36.6 ± 22.3 ^δ^	Enzymatic detoxification, rennal excretion	4-HNE: Reduced after HD session	
4-hydroxy-2-hexenal (4-HHE), μg/L	25.1 ± 9.9	63.8 ± 25.3 ^δ^			
4-hydroxy-2-nonenal (4-HNE), μg/L	16.4 ± 9.0	117.3 ± 47.7 ^δ^			
4-hydroxy-octenal, μg/L	10.7 ± 3.6	27.8 ± 13.8 ^δ^			
**Arachidonic Acid By-Products of Lipid Peroxidation**			Renal excretion, Enzymatic detoxification	No change	[119,122,123,124,125,126,127,128]
Total F_2_-isoprostanes, pg/mL *	162 ± 73	270 ± 10 ^δ^			
Unesterified F_2_-isoprostanes, pg/mL	37.6 ± 17.2	96.2 ± 48.8 ^δ^			
Esterified F_2_-isoprostanes, pg/mL	146.8 ± 58.4	220.4 ± 154.8 ^δ^			
**Lipoprotein Products**					
ApoB_48_ level, mg/L	3.7 ± 2.3	19.3 ± 13.9 ^δ^			[33]
Oxidized LDL, mg/L	0.22 ± 0.05	1.92 ± 0.29 ^δ^	Accumulation in atherosclerotic lesions	Increased after HD session	[53,54,55,56]
3-chlorotyrosine, μmol/mol of tyrosine	<0.3	3.5 ± 0.5 ^δ^	-	-	[64]
Lp(a) level, mg/dL	18.4 ± 22.8	23.4 ± 34.6 ^δ^	Renal and hepatic clearance	No changes or increased after HD session	[56,109,110,111]

Data are expressed as means ± SD. * computed from the data available in the original article, ^δ^ *p* < 0.05 vs. control; Lp(a): lipoprotein A, PUFAs: polyunsaturated fatty acids.

## Abbreviations

ABCA-1	ATP-biding cassette 1
ACAT	acetyl-CoA acetyltransferase-1
AGEs	advanced glycation end-products
ALEs	advanced lipoxidation end-products
ApoA/B/C/E	apolipoprotein A/B/C/E
CETP	cholesterol-ester transfer protein
CKD	chronic kidney disease
cLDL	carbamylated low-density lipoprotein
CV	cardiovascular
CVD	cardiovascular disease
ESRD	end-stage renal disease
EuTox	European uremic toxin work group
FAPPs	fatty acid peroxidation products
GFR	glomerular filtration rate
GPX	gluthatione peroxidase
HD	hemodialysis
HDL	high density lipoproteins
4-HNE	4-hydroxy-2-nonenal
4-HHE	4-hydroxy-2-hexenal
HOCL	hypochlorous acid
ICAM-1	intercellular adhesion molecule 1
IL-1ß	interleukine 1ß
LCAT	lecithin-cholesterol acyltransferase
LCFA	long chain fatty acids
LDL	low-density lipoproteins
LOX-1	lectin-like oxidized lox density receptor 1
Lp(a)	lipoprotein a
LRP	LDL receptor protein
LVH	left ventricle hypertrophy
MCP-1	monocyte chemoattractant protein 1
MDA	malondialdehyde
MPO	myeloperoxidase
NO	nitric oxide
oxPLs	oxidized phospholipids
oxLDL	oxidized low-density lipoproteins
PLs	phospholipids
PON1	paraoxonase 1
PTMDPs	post translational modification derived products
PUFAs	polyunsaturated fatty acids
RAS	renin-angiotensin system
RNS	reactive nitrogen species
ROS	reactive oxygen species
SOD	superoxide dismutase
SR-B1	scavenger receptor class B member 1
TBARS	thiobarbituric acid reactive species
TGRL	triglyceride-rich lipoproteins
TNF-α	tumor necrosis factor α
TxA2-R	thromboxane A2 receptor
VCAM-1	vascular cell adhesion molecule 1
